# Lotus Bee Pollen Extract Inhibits Isoproterenol-Induced Hypertrophy via JAK2/STAT3 Signaling Pathway in Rat H9c2 Cells

**DOI:** 10.3390/antiox12010088

**Published:** 2022-12-30

**Authors:** Shuo Han, Lifu Chen, Yi Zhang, Shihui Xie, Jiali Yang, Songkun Su, Hong Yao, Peiying Shi

**Affiliations:** 1 Department of Traditional Chinese Medicine Resource and Bee Products, College of Animal Sciences (College of Bee Science), Fujian Agriculture and Forestry University, Fuzhou 350002, China; 2 Department of Pharmaceutical Analysis, School of Pharmacy, Fujian Medical University, Fuzhou 350122, China; 3 State and Local Joint Engineering Laboratory of Natural Biotoxins, Fujian Agriculture and Forestry University, Fuzhou 350002, China

**Keywords:** lotus bee pollen extract, cardiomyocyte hypertrophy, JAK2/STAT3 signaling pathway, oxidative stress, inflammation

## Abstract

Bee pollen possesses an anti-cardiomyocyte injury effect by reducing oxidative stress levels and inhibiting inflammatory response and apoptosis, but the possible effect mechanism has rarely been reported. This paper explores the effect of the extract of lotus bee pollen (LBPE) on cardiomyocyte hypertrophy (CH) and its mechanism. The main components of LBPE were identified via UPLC-QTOF MS. An isoproterenol-induced rat H9c2 CH model was subsequently used to evaluate the protection of LBPE on cells. LBPE (100, 250 and 500 μg∙mL^−1^) reduced the surface area, total protein content and MDA content, and increased SOD activity and GSH content in CH model in a dose-dependent manner. Meanwhile, quantitative real-time PCR trials confirmed that LBPE reduced the gene expression levels of CH markers, pro-inflammatory cytokines and pro-apoptosis factors, and increased the Bcl-2 mRNA expression and Bcl-2/Bax ratio in a dose-dependent manner. Furthermore, target fishing, bioinformatics analysis and molecular docking suggested JAK2 could be a pivotal target protein for the main active ingredients in the LBPE against CH. Ultimately, Western blot (WB) trials confirmed that LBPE can dose-dependently inhibit the phosphorylation of JAK2 and STAT3. The results show that LBPE can protect against ISO-induced CH, possibly via targeting the JAK2/STAT3 pathway, also suggesting that LBPE may be a promising candidate against CH.

## 1. Introduction

Cardiac hypertrophy is an adaptive response to many cardiovascular diseases such as hypertension, myocardial ischemia, coronary heart disease and heart failure (HF) [1]. Cardiac hypertrophy is accompanied by an expansion in cardiomyocyte volume and an increase in blood volume during the initial phase of adaptation, and this effect is considered beneficial. However, under the pathological circumstance, along with sustained pressure overload, the capillaries cannot achieve the requirements of nutrition and oxygen in the heart, which leads to the transformation of cardiac hypertrophy from adaptive to pathological [1,2]. Besides causing cardiac dysfunction, cardiac hypertrophy is also a potential independent factor of HF, which has a high rate of disability and mortality [3]. Hence, treating or suppressing cardiac hypertrophy is identified as a crucial target for preventing HF.

Isoproterenol (ISO), a β-adrenergic receptor (β-AR) agonist, has been widely applied to establish the cardiomyocyte hypertrophy (CH) model and could be used for evaluating the protective effects of potential medicines [4,5]. The activation of β-adrenergic signaling in the heart contributes to the hypertrophic phenotype and induces many mechanisms such as enhanced protein synthesis, elevated oxidative stress, etc. [5].

Bee pollen is a kind of medicinal food collected from the stamens of plants and processed into pollen masses by bees. The major components of bee pollen contain nucleosides, carbohydrates, flavonoids, phenolic acids, polyamines, amino acids and other active ingredients [6,7]. Bee pollen possesses potential pharmacological properties such as antioxidative, anti-inflammatory, antibacterial and anticancer, etc. [8]. A recent review has summarized the clinical trials and patent applications of bee pollen, and found that bee pollen provided protection against many diseases, including cardiovascular diseases [9]. *Schisandra Chinensis* bee pollen extract and rape bee pollen extract have been reported to protect against H9c2 cardiomyocyte injuries by reducing oxidative stress levels and inhibiting inflammatory response and apoptosis [4,7], but the possible effect mechanism has rarely been reported. Up to nowadays, there is not any report on the effect of lotus bee pollen extract (LBPE) against cell injury, which is unbeneficial to the development and utilization of the medicinal food.

The Janus kinase (JAK)/signal transducer and activator of transcription (STAT) pathway is a way of transmitting external signals to the nucleus, among which JAK2/STAT3 signal transduction is a crucial pathway related to CH [10]. When receiving external stimulation, the cytokines bind to their corresponding receptor and activate JAK2. Activated JAK2 phosphorylates and dimerizes nearby STAT3 in the cytoplasm. The dissociated dimer is transferred to the nucleus and participates in cell proliferation, differentiation and apoptosis by inducing the encoding of targeted DNA genes [11]. It has been evidenced that the suppression of the JAK2/STAT3 signaling pathway could play a prohibitive effect on myocardial hypertrophic injury [12,13]. STAT3 is a signal transduction protein that is considered as an essential mediator in cardiac remodeling. In myocardial infarction, STAT3 is a stress protein of myocardial injury, and unrestricted activation of STAT3 is detrimental. During this process, the increased level of interleukin-6 (IL-6) in serum is considered to be the prognostic marker of myocardial infarction and HF [14]. Previous research verified that the stimulation of β-AR upregulated the expression of the IL-6 cytokine family, especially IL-6 that dominates the phosphorylation of STAT3 in which gp130 intervenes [15]. According to the reports above-mentioned, it can reasonably be deduced that the effect mechanism of bee pollen against cell injury could refer to targeting the IL-6 and JAK2/STAT3 pathways.

In this paper, the protective effect of LBPE against ISO-induced CH was evaluated. Meanwhile, target fishing, bioinformatics analysis and molecular docking suggested JAK2 could be a pivotal target protein. The possibly involved JAK2/STAT3 pathway was validated using Western blot, and the JAK2 phosphorylation was blocked by AG490 (an inhibitor for JAK2). The present study for the first time provides scientific evidence of LBPE against cardiac hypertrophy and confirms the possible effect target and pathway.

## 2. Materials and Methods

### 2.1. Chemicals and Reagents

Lotus bee pollen was purchased from the Mulan Mountain Bee Farm in Wuhan, China in December 2018. The lotus bee pollen with a purity greater than 95% was identified according to GB-31636-2016 by associated Professor Peiying Shi and stored in 4 °C at the traditional Chinese medicine pharmacology laboratory of Fujian Agriculture and Forestry University. Methanol (HPLC-grade) was purchased from Merck (Darmstadt, Germany). Formic acid, anhydrous ethanol and phosphate buffer solution PBS were purchased from Sinopharm Chemical Reagent Co., LTD. Uridine, adenosine, guanosine (purity ≥ 98%), captopril and cell-grade DMSO were purchased from Solarbio (Beijing, China). Fetal bovine serum (FBS) and Dulbecco’s modified Eagle medium (DMEM, high glucose) were purchased from Cellmax (Beijing, China). Trypsin enzyme and 1% penicillin-streptomycin were purchased from HyClone (Logan, UT, USA). ISO was purchased from Aladdin (Shanghai, China). The BCA protein quantitative assay kit and SDS-PAGE gel preparation kit were purchased from Beyotime (Shanghai, China). MDA, GSH and SOD kits were provided by Nanjing Jiancheng Bioengineering Institute (Nanjing, China). YF^®^488-Phalloidin was provided by US Everbright Inc (Suzhou, China). The Trans Zol UP kit was purchased from TransGen Biotech (Beijing, China). Reverse transcription and fluorescence quantitative PCR kits were purchased from Vazyme (Nanjing, China). PCR primers were designed by Fuzhou Shangya Biotechnology Co., Ltd. (Fuzhou, China). AG490 was provided by GLPBIO (Montclair, USA). Antibodies against JAK2, STAT3 and glyceraldehyde-3-phosphate dehydrogenase (GAPDH) were purchased from ABclonal (Wuhan, China). The antibody against p-JAK2 was provided by Abcam (Shanghai, China), the antibody against p-STAT3 was purchased from Cell Signaling Technology (Beverly, USA), and the antibody against Caspase-3 and TNF-α were purchased from Yeasen Biotechnology Co., Ltd. (Shanghai, China). Skim milk powder was purchased from Saiguo Bio Co., Ltd. (Guangzhou, China).

### 2.2. LBPE Preparation and Ultra-Performance Liquid Chromatography–Quadrupole Time-of-Flight Mass Spectrometry (UPLC-QTOF MS) Analysis

LBPE was extracted according to our previous research [7]. The dried bee pollen was pulverized into powder and then extracted with 70% ethanol at a ratio of 1:15 (*w*/*v*). Combining shaking with shaker for 24 h and ultrasonic extraction for 20 min, repeating for twice, the resulting liquid was centrifuged for 10 min at 7104 g and 4 °C. After evaporation of the supernatant to dispose of the ethanol, the LBPE was obtained via freeze-drying.

To identify the main ingredients, 10 mg of LBPE was dissolved in 1 mL of 70% methanol. After vortexing for 2 min and ultrasonic extraction for 5 min, the sample was centrifuged at 12704 g for 10 min. The resulting supernatant was transferred to an injection vial for UPLC-QTOF MS analysis. The UPLC-QTOF MS analysis condition was identical with our previous report [4], and can be seen in the Supplementary Data.

### 2.3. Cell Culture and Group Administration

The H9c2 (2-1) (Procell CL-0089) myocardial cell line was purchased from Procell Life Science & Technology Co., Ltd. (Wuhan, China). The cells were inoculated in a culture bottle and maintained in high-glucose DMEM supplemented with 10% FBS and 1% antibiotics (penicillin–streptomycin mixture) at 37 °C in a 5% CO_2_ incubator (C150, Binder).

After incubation for 24 h, the cultured cells were divided into six groups: negative control, ISO (50 μmol∙L^−1^), positive control (0.1 mmol∙L^−1^ captopril), low concentration of LBPE (100 μg∙mL^−1^ LBPE), middle concentration of LBPE (250 μg∙mL^−1^ LBPE), and high concentration of LBPE (500 μg∙mL^−1^ LBPE). First, the negative control and ISO groups were cultured with high-glucose complete medium for 24 h, and the positive control and LBPE groups were treated with 0.1 mmol∙L^−1^ captopril and 100, 250 and 500 μg∙mL^−1^ LBPE (obtained from captopril and LBPE stock solution diluted with high glucose complete medium), respectively, for 24 h. Then all the groups were washed with PBS and starved for 12 h, and the cells were damaged with 50 μmol∙L^−1^ ISO (obtained from ISO stock solution diluted with high-glucose complete medium) for 48 h, except for the negative control group, which was cultured with high-glucose complete medium for 48 h. The samples were used in subsequent experiments.

### 2.4. Morphological Analysis

The cells were fixed and stained according to the YF^®^488 labeled Phalloidin staining instruction. The treated cells were fixed in 4% paraformaldehyde solution on ice for 15 min, then cleaned with PBS. After permeation with 0.5% Triton X-100 solution for 10 min and cleaning with PBS, the cells were stained for 30 min with dye liquor prepared in advance (5 μL YF^®^488 labeled Phalloidin diluted with 200 μL PBS). Changes in cell morphology were observed under a 400× inverted fluorescence microscope after washing away the dye with PBS. Four to six views were selected randomly from pictures of each sample, and the surface areas were measured with Oplenic software (Hangzhou, China).

### 2.5. Determination of Cellular Antioxidant Capacity and Total Protein Content

The total protein content, GSH, SOD and MDA levels were determined using test kits based on the manufacturer’s instructions described with minor modifications. Briefly, after treatment, the H9c2 cells were collected by centrifuging at 137 g for 5 min and then 0.3 mL of normal saline was added to the precipitation for total protein content, GSH, SOD assay and 0.5 mL Reagent five for MDA assay. After electric grinding for 15–20 s at 30 s intervals (ground four times in total) under an ice bath, the resulting suspension was collected for determination. The following experiments were operated according to the manufacturer’s instructions.

### 2.6. Quantitative Real-Time PCR

RNA was extracted according to the TransZol UP kit, and the qualities of the RNA were determined. Reverse transcription was performed via the HiScript^®^ Ⅱ Q RT SuperMix kit in line with the manufacturer’s instruction to obtain the required cDNA. Then the qPCR was carried out using the ChamQ Universal SYBR qPCR Master Mix kit according to instruction, and each sample was equipped with 3 multiple wells. For qPCR, the initial denaturation was fulfilled for 3 min at 95 °C, followed by 40 cycles annealing for 10 s at 95 °C and 30 s at 59 °C with subsequent melting curve analysis, and then increasing the temperature from 65 °C to 95 °C with a rate of 0.5 °C per 5 s. The mRNA expression levels of ANP, BNP, β-MHC, IL-6, TNF-α, Bax, Bcl-2, Caspase-3, Caspase-8 and Caspase-9 in H9c2 cells were calculated relatively according to that of GAPDH with the 2^−ΔΔCt^ method. Following primer design principles, PCR primers were designed through the NCBI web page and synthesized and purified by Fuzhou Shangya Biotechnology Co., Ltd. (Table 1).

### 2.7. Target Fishing, Bioinformatic Analysis and Molecular Docking

Target fishing was performed using PharmMapper, a web on-line server (http://lilab-ecust.cn/pharmmapper/, available on 12 September 2022) according to previous reports [16,17,18]. Briefly, the molecular structures of the five ingredients, I3XG, gluconic acid, adenosine, uridine and guanosine were built up using Sybyl-X 1.3 and optimized using MMFF94 minimization, respectively, followed by saving as Mol2 files. Each Mol2 file was submitted to PharmMapper to search the potential target proteins. The top 300 proteins in light of the Fit score order were reserved, and the matched proteins with a zscore > 0 were ultimately screened for the next study.

To predict the possible targets against cardiovascular diseases for LBPE, DOSE analysis was originally performed using R package ”DOSE” with the corresponding genes of the above-screened proteins as the input datasets. Those genes that were enriched in cardiovascular diseases, such as arteriosclerotic cardiovascular disease, arteriosclerosis, coronary artery disease and myocardial infarction, were pooled to further perform PPI analysis using the STRING database (https://cn.string-db.org/, accessed on 20 September 2022) and the result was visible via Cytoscape 3.6.0. The genes with high interaction scores and high relevance to CH were screened for the next study.

For molecular docking, the corresponding proteins of the genes above-screened were downloaded from RCSB Protein Data Bank (https://www.pdbus.org/, accessed on 20 September 2022). Molecular docking between protein and individual ingredient (I3XG, gluconic acid, adenosine, uridine or guanosine) was carried out using LeDock software (Lephar Research, Stockholm, Sweden) (www.lephar.com, accessed on 20 September 2022) and the results were visible via PyMOL 2.5 (Schrödinger LLC).

### 2.8. Blocking p-JAK2 by AG490

Refer to *2.3*, H9c2 cells were cultivated and divided into different treatment groups: negative control, ISO (50 μmol∙L^−1^), high concentration of LBPE (500 μg∙mL^−1^ LBPE), AG490 (10 μmol∙L^−1^), and LBPE + AG490 (500 μg∙mL^−1^ LBPE + 10 μmol∙L^−1^ AG490). Cells were incubated with AG490 for 30 min before ISO injury to verify the effects of JAK2-blockage on ISO-induced CH. Then the cells were collected for RT-qPCR assay to determine the changes in the gene expression level of a CH marker, ANP.

### 2.9. Western Blot

The thawed samples were homogenized in total protein extraction buffer supplemented with protease and phosphatase inhibitors. After determination of protein concentration using BCA protein assay kit, equal amounts of protein were denatured. Loaded to each lane were 40 μg protein samples (for detecting p-JAK2, JAK2, p-STAT3, STAT3), or 15 μg protein samples (for detecting TNF-α and Caspase-3); they were separated via 10% SDS-PAGE. With the effect of the electric field, the protein was transferred to the polyvinylidene fluoride membranes. The membranes were blocked by skim milk and probed with primary antibodies (p-JAK2, JAK2, p-STAT3, STAT3, TNF-α, Caspase-3) at 1:1000 dilution and GAPDH at 1:2500 dilution overnight at 4 °C, followed by a washing step, and the membranes were detected with secondary rabbit polyclonal antibodies for 1 h. After washing with TBST, the target bands were exposed to the film with the help of a chemiluminescence solution. The variation of levels was analyzed using Image J 1.53t software (National Institutes of Health, Bethesda, MD, USA).

### 2.10. Statistical Analysis

All data are expressed as mean ± standard error and the Statistical Product and Service Solutions (SPSS) 19.0 software package for Windows (International Business Machines Corporation, Armonk, NY, USA) was used for one-way ANOVA to analyze the significant differences among samples. Values of *p* less than 0.05 were considered statistically significant between groups. All experiments were repeated at least three times.

## 3. Results

### 3.1. Identification of the Main Components in LBPE

**Figure 1:**
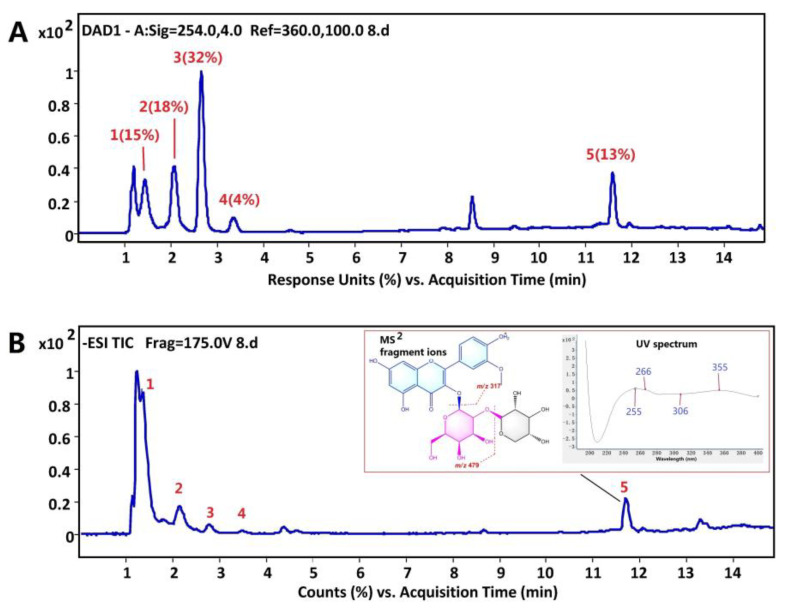
(**A**) UV chromatogram at 254 nm and (**B**) total ion chromatogram in negative mode of LBPE with the insert graphs of (+)MS^2^ fragment ions and UV spectrum of peak 5.

The UV chromatogram at 254 nm and total ion current chromatogram in the negative ion mode of LBPE are shown in Figure 1. The retention time (t_R_), molecular weight, double bond equivalences (DBE) and formula are summarized in Table S1. Five main components were identified or preliminarily characterized. Peak 1 (occupying 15% according to the peak area normalization method in the UV chromatogram) was identified as gluconic acid according to our previous report [7]. Peaks 2 (18%), 3 (32%) and 4 (4%) were identified as uridine, adenosine and guanosine, respectively, by comparing with the reference compounds. Peak 5 shows a [M-H]^−^ ion at *m/z* 609.14676, which is accordant with the theoretical molecular weight of C_27_H_30_O_16_. The (+) MS^2^ spectrum shows two fragment ions at *m/z* 479 and 317, which are assigned to [M+H−132]^+^ and [M+H−132−162]^+^, maybe corresponding to the neutral loss of xylosyl (or arabinosyl) and xylosyl (or arabinosyl) plus galactosyl (or glucosyl). The UV spectrum of Peak 5 (see the insert graph of Figure 1B) shows maximum lengths at 255, 266, 306 and 355 nm, which are completely consistent with the spectrum properties of a flavone diglycoside, isorhamnetin 3-xylosyl-(1→2)-galactopyranoside from *Prunus padus* L. flowers [19]. Accordingly, Peak 5 was tentatively identified as isorhamnetin 3-xylosyl-(1→2)-galactopyranoside (I3XG).

### 3.2. Effects of LBPE on the Morphology, Surface Area and Total Protein Content of H9c2 Cells Induced by ISO

**Figure 2:**
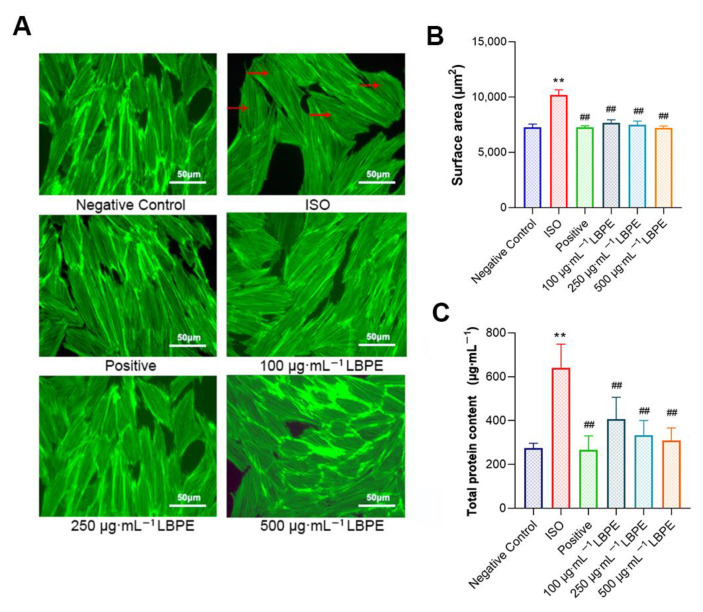
Effects of LBPE on (**A**) the morphology using phalloidin staining (×400), (**B**) surface area and (**C**) total protein content of H9c2 cardiomyocytes (n = 6). “→” indicates that the cells lose their spindle shape. ISO group vs. negative control group, ** *p* < 0.01. Positive and LBPE groups vs. ISO group, ## *p* < 0.01.

As shown in Figure 2A, the cells in the negative control group exhibited normal shuttle-shaped morphology, while in the ISO group, the cells were obviously hypertrophic, and some cells lost their spindle shape; additionally, the intercellular space decreased, the distribution was uneven and disordered, and it was easier to gather the cells into clusters. By contrast, in the positive and LBPE groups, the intercellular space became wider, with the sizes of cells reduced, and the form of the cells was restored to the spindle. The results of cell surface area after cytoskeleton staining are shown in Figure 2B. Compared with the negative control group, the cell surface area of the ISO group increased by 40.28% on average. With the administration of LBPE and compared to the ISO group, the damage was decreased by 28.52% (100 µg∙mL^−1^), 29.82% (250 µg∙mL^−1^) and 30.40% (500 µg∙mL^−1^) in different concentrations, respectively. Meanwhile, the cell surface area of LBPE groups increased by only 7.05% (100 µg∙mL^−1^), 5.11% (250 µg∙mL^−1^) and 4.23% (500 µg∙mL^−1^) compared to the negative control group, and there was no significant difference between negative control group and LBPE groups. These results suggest that LBPE can effectively inhibit the occurrence of CH.

The hypertrophic response of rat cardiomyocytes also included the increased total protein synthesis [20]. The results of total protein content are shown in Figure 2C. The total protein content in the ISO group was much higher than that of the negative control group (*p* < 0.01), while this increase was significantly suppressed by LBPE (*p* < 0.01).

### 3.3. Effect of LBPE on ANP, BNP and β-MHC mRNA Expression Levels in rat H9c2 Cells Injured by ISO

**Figure 3:**
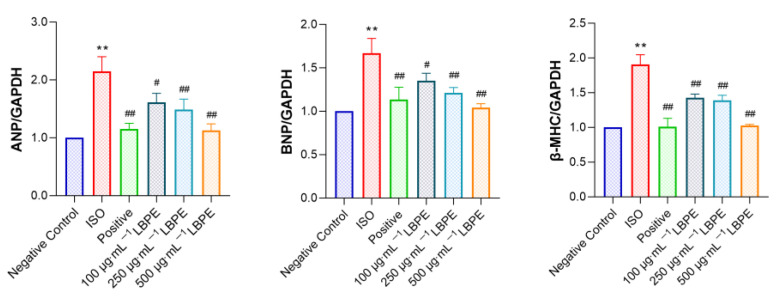
Effects of LBPE on the gene expression of hypertrophy markers in H9c2 cardiomyocytes (n = 3). ISO group vs. negative control group, ** *p* < 0.01. Positive and LBPE groups vs. ISO group, # *p* < 0.05, ## *p* < 0.01.

As shown in Figure 3, compared with the negative control group, the mRNA expression levels of ANP, BNP and β-MHC in the ISO group significantly increased (*p* < 0.01), which indicates the success of establishing the CH model. By contrast, the mRNA expression levels of these CH markers were inhibited by LBPE (*p* < 0.05, or *p* < 0.01), and show dose-dependency. These results also demonstrate that LBPE alleviated ISO-induced CH.

### 3.4. Effects of LBPE on MDA, SOD and GSH Levels in H9c2 Cells Injured by ISO

As shown in Table 2, ISO treatment significantly decreased GSH and SOD levels and increased the production of MDA in cultured cells (*p* < 0.01). Compared with the ISO group, the levels of GSH and SOD increased in LBPE groups, and in particular, the 500 µg∙mL^−1^ LBPE group demonstrated a significant difference (*p* < 0.05, *p* < 0.01), as well as the content of MDA significantly decreasing in LBPE groups (*p* < 0.05, *p* < 0.01). All of them presented a certain dose-dependency. These results reveal that LBPE can reduce the oxidative damage induced by ISO to H9c2 cells and improve the antioxidant capacity of cells.

### 3.5. Effects of LBPE on Inflammation in H9c2 Cells Injured by ISO

**Figure 4:**
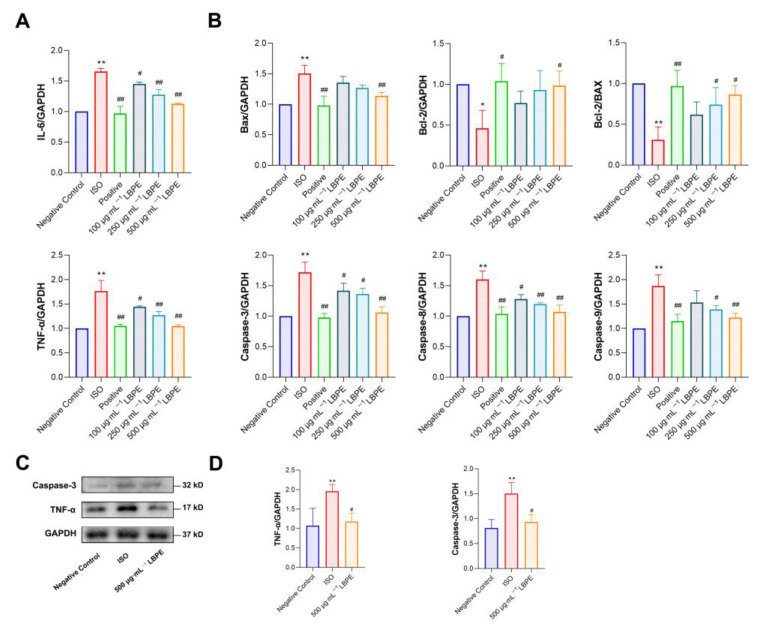
Effects of LBPE on the gene expression of (**A**) pro-inflammatory cytokines and (**B**) apoptosis-related factors, as well as (**C**) the protein expression and (**D**) quantified Western blot results of TNF-α and Caspase-3 in H9c2 cardiomyocytes (n = 3). ISO group vs. negative control group, * *p* < 0.05, ** *p* < 0.01. Positive and LBPE groups vs. ISO group, # *p* < 0.05, ## *p* < 0.01.

As shown in Figure 4A, the mRNA expression levels of IL-6 and TNF-α significantly increased in the ISO group (*p* < 0.01), which were inhibited significantly in LBPE groups (*p* < 0.05, or *p* < 0.01). The effect was particularly remarkable in the high-concentration group. In addition, the protein expression level of TNF-α significantly increased in the ISO group (*p* < 0.01) and was inhibited significantly in the 500 µg∙mL^−1^ LBPE group (*p* < 0.05), shown in Figure 4C,D. These results indicate that LBPE pre-treatment can significantly suppress the inflammatory response caused by ISO and improve the anti-inflammatory ability of the cells.

### 3.6. Effects of LBPE on Apoptosis in H9c2 Cells Injured by ISO

As shown in Figure 4B, compared with the negative control group, the mRNA expression levels of Bax, Caspase-3, Caspase-9 and Caspase-8 increased significantly (*p* < 0.01), while those of anti-apoptotic factor Bcl-2 and the Bcl-2/Bax ratio decreased significantly in the ISO group (*p* < 0.05, *p* < 0.01). After treatment with LBPE, compared with the ISO group, the mRNA expression levels of Bax, Caspase-3, Caspase-9 and Caspase-8 decreased significantly (*p* < 0.05, or *p* < 0.01), and the expression of Bcl-2 and the Bcl-2/Bax ratio increased significantly (*p* < 0.05). A high concentration of LBPE (500 µg∙mL^−1^) even demonstrated a similar effect to the positive group. Furthermore, the protein expression level of Caspase-3 significantly increased in the ISO group (*p* < 0.01) and was inhibited significantly in the 500 µg∙mL^−1^ LBPE group (*p* < 0.05), shown in Figure 4C,D. These results suggest that LBPE can relieve ISO-induced hypertrophic injury by inhibiting apoptosis.

### 3.7. Targets and Effect Pathways Prediction for LBPE against CH

**Figure 5:**
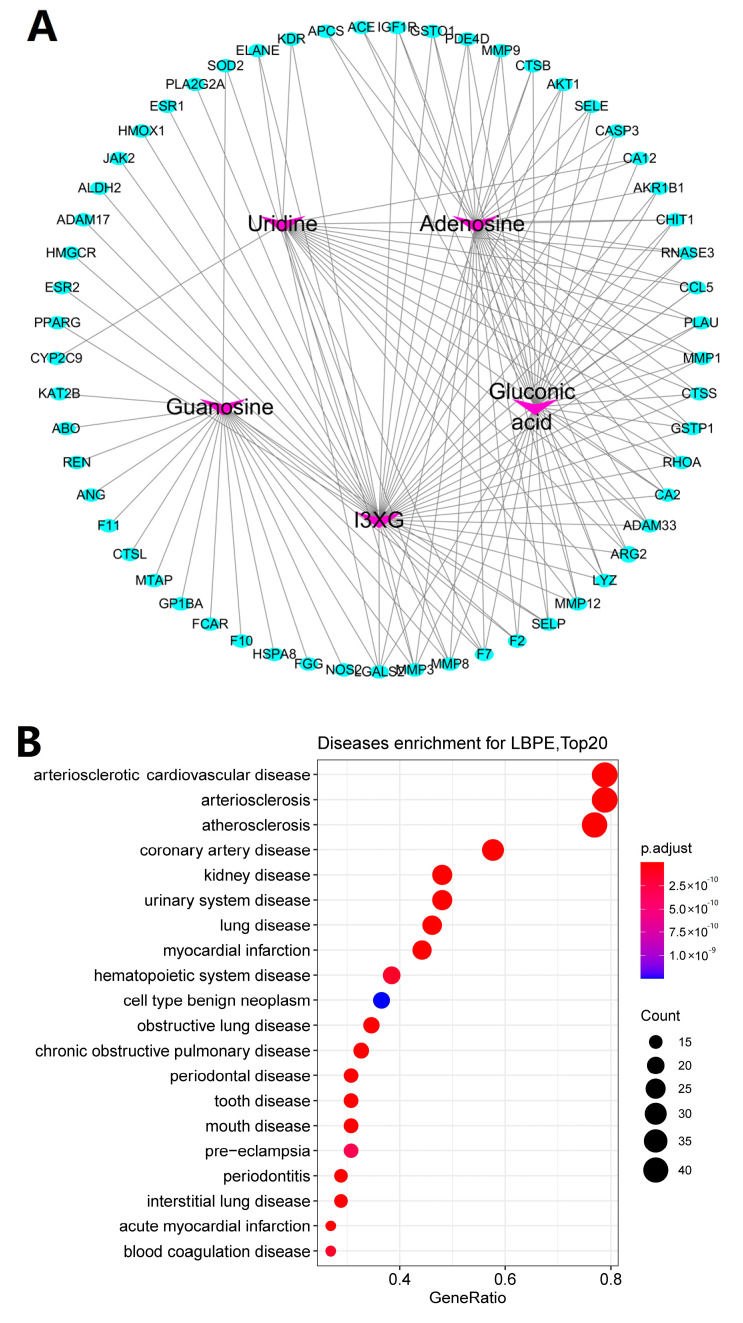

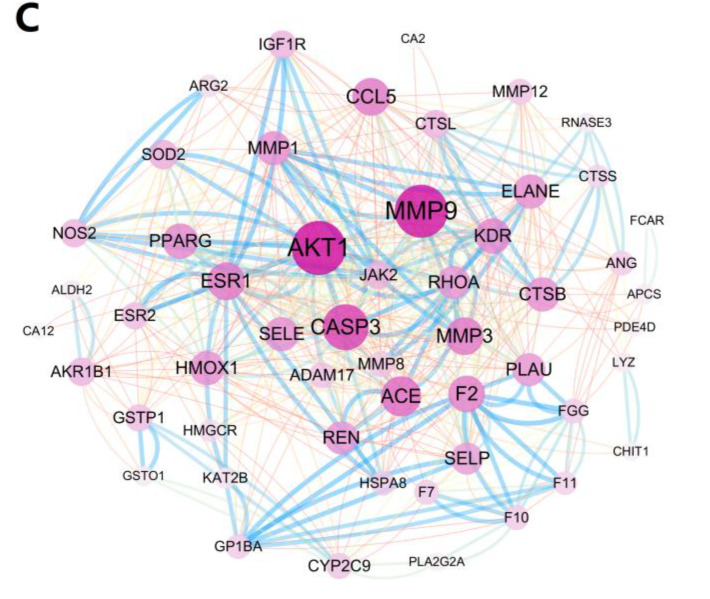
Graphs of (**A**) component–target network, (**B**) DO enrichment analysis and (**C**) PPI analysis.

**Figure 6:**
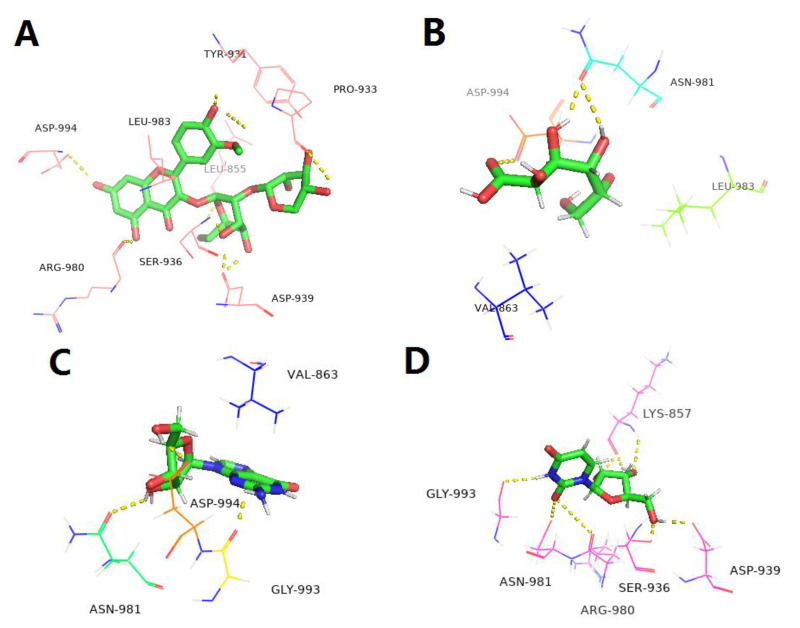
Molecular docking results of JAK2 (PDB: 2B7A) with (**A**) I3XG, (**B**) gluconic acid, (**C**) guanosine and (**D**) uridine.

As shown in Figure 5A,B, 57 proteins were screened with a z-score of >0 and predicted to be related to cardiovascular disease. PPI analysis (Figure 5C) shows that proteins AKT1, MMP3 and CASP3 with high node degrees (32, 31 and 25, respectively) and JAK2 with high combined scores (>0.9) with the neighboring nodes, NOS2 and IGF1R, are located in the center of the network graph, suggesting the possible contribution of these proteins in exerting the therapeutic effects of LBPE. In particular, considering JAK2 a crucial target related to CH according to previous reports [10], molecular docking was further performed between the protein and the individual ingredient (I3XG, gluconic acid, adenosine, uridine and guanosine). As shown in Figure 6, except for adenosine, the other four ingredients I3XG, gluconic acid, uridine and guanosine have favorable docking affinity energy with JAK2 (−9.35, −4.63, −7.30 and −5.56 kcal mol^−1^), which further suggests the target role of JAK2 for the therapeutic effects of LBPE against CH.

### 3.8. Effect of LBPE on JAK2/STAT3 Signal Pathway in H9c2 Cells

**Figure 7:**
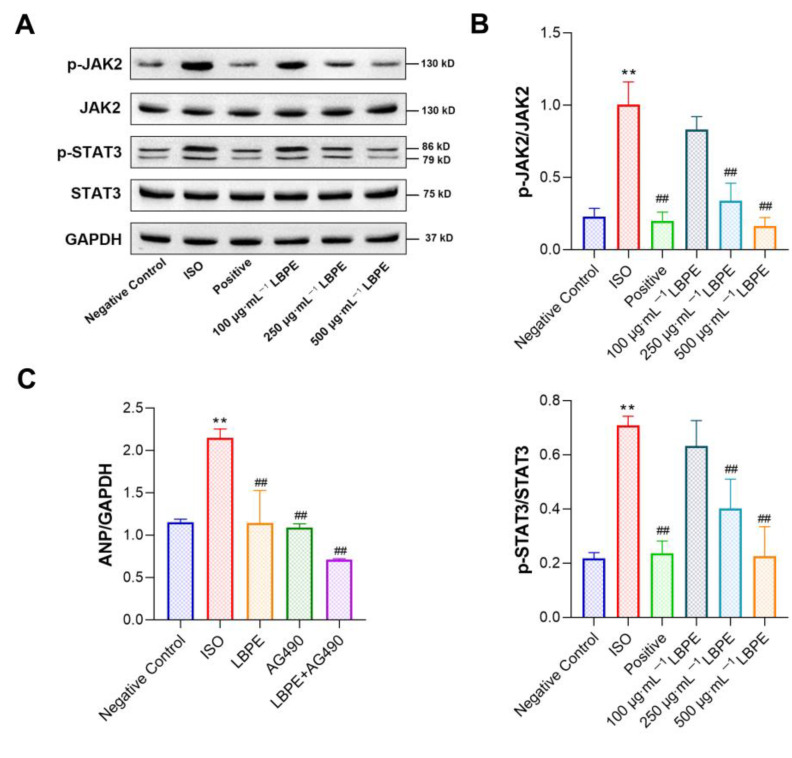
Effects of LBPE on JAK2/STAT3 signaling pathway in H9c2 cardiomyocytes (n = 3). (**A**) Protein expression was measured using Western blot. (**B**) Quantified Western blot results of JAK2, p-JAK2, STAT3 and p-STAT3. (**C**) Gene expression of ANP when treated with LBPE (500 μg∙mL^−1^) and/or AG490 (10 μmol∙L^−1^). ISO group vs. negative control group, ** *p* < 0.01. Positive and LBPE groups vs. ISO group, ## *p* < 0.01.

To further verify the mechanism of LBPE on ISO-induced CH, we detected the protein expression levels of JAK2, p-JAK2, STAT3 and p-STAT3 in differently treated cells (Figure 7A,B). Compared with the negative control group, the phosphorylation levels of JAK2 and STAT3 were significantly enhanced in the ISO group (*p* < 0.01), and this increase was inhibited by LBPE in a dose-dependent manner, in particular significantly reversed by 250 µg∙mL^−1^ and 500 µg∙mL^−1^ LBPE (*p* < 0.01) in a dose-dependent manner. Additionally, the total protein contents of JAK2 and STAT3 were unchanged.

In addition, to evaluate whether JAK2 phosphorylation is crucial to the anti-CH effect of LBPE, we abrogated JAK2 phosphorylation using AG490 (a well-known inhibitor for JAK2). As shown in Figure 7C, compared with the ISO group, similar to LBPE, application of AG490 alone significantly reduced the mRNA expression levels of ANP (*p* < 0.01), and combined application of LBPE and AG490 resulted in enhanced decrease in the mRNA expression levels of this CH marker (*p* < 0.01).

These results indicate that LBPE could downregulate the JAK2/STAT3 signaling pathway to ameliorate ISO-induced CH in H9c2 cells, which also further validates the target role of JAK2 for the therapeutic effects of LBPE against CH.

## 4. Discussion

The main components of LBPE, including a flavone diglycoside (I3XG), a carbohydrate (gluconic acid) and three nucleosides (adenosine, uridine and guanosine) were identified via UPLC-QTOF MS. I3XG is a rare flavone diglycoside that has only ever been identified in *Prunus padus* L. flowers [19]. It is the first time that the ingredient I3XG has been identified with a high relative content (13%) in bee pollen. The target fishing, bioinformatics analysis and molecular docking also give promising prediction on the therapeutic effect of I3XG against CH, which of course require further and more validation experiments. Adenosine has anti-hypertrophic and anti-adrenergic functions, and adenosine A1 receptors could be considered as potential targets for therapeutic strategies to prevent transition from compensated myocardial hypertrophy to decompensated HF due to chronic cardiac pressure overload [21]. The uridine reactive purinergic P2Y6 receptor (P2Y6R) contributes to the development of cardiovascular remodeling in rodents, and the loss of P2Y6R in mice that was observed could promote pathological CH induced by ISO [22]. However, whether the other main ingredients, such as glucose acid and guanosine, have a positive contribution to the anti-CH effect of LBPE, there is still a lack of reports, which requires further investigation. Anyway, these previous research papers provided the possibility that LBPE could play a protective effect on CH induced by ISO.

In the context of current social life, people are often burdened with multiple pressures. Chronic stress causes increased secretion of catecholamines and stimulates β-AR, which induces cardiac burden [23]. Additionally, the incidence of cardiovascular disease will increase at the same time. ISO, as a β-adrenergic agonist, is applicable for the stress model. Many researchers have determined that ANP, BNP and β-MHC can be used as biomarkers to evaluate CH [24]. In this study, after treatment of ISO onto H9c2 cells, the up-regulated mRNA expression levels of these hypertrophic biomarkers, the increased total protein content and the result of cytoskeleton staining indicated the occurrence of CH. With the pre-treatment of LBPE, the above hypertrophic features were ameliorated. These results preliminarily suggest that LBPE could inhibit CH induced by ISO in H9c2 myocardial cells.

The JAK2/STAT3 signaling pathway is a common signal transduction pathway in the development of HF and plays a crucial role in myocardial hypertrophy [25]. JAK2 could regulate cardiac contractility and promote the transition from cardiac hypertrophy to cardiac dysfunction. JAK2-inhibitor tyrphostin AG490 treatment alleviated CH induced by stress [26]. STAT proteins are involved in a variety of cellular activities, such as proliferation, differentiation, apoptosis and angiogenesis. The activation of STAT3 in cardiomyocytes is associated with the upregulation of multiple target genes [27]. Recently, research has emphasized that β-ARs can directly mediate the activation of STAT3, which is an important regulator in CH [28]. Celastrol attenuates angiotensin II-induced cardiac remodeling by targeting STAT3 [29]. Protocatechuic aldehyde protects against ISO-induced cardiac hypertrophy via inhibition of the JAK2/STAT3 signaling pathway [10]. These studies indicate that negative regulation of the JAK2/STAT3 pathway is expected to be a promising therapeutic strategy for myocardial remodeling. In the present study, target fishing, DOSE, PPI analysis and molecular docking predict that JAK2 is a pivotal target for the main ingredients in LBPE. The phosphorylation levels of JAK2 and STAT3 were elevated in ISO-induced H9c2 myocardial cells, which were indeed inhibited by LBPE. JAK2 inhibitor AG490 confirmed that inhibition of JAK2 enhanced the anti-CH effect of LBPE. These results confirm the target role of JAK2 for the therapeutic effects of LBPE against CH.

Inflammation predominantly mediates the progression of CH [1]. Various reasons such as stress overload and oxidative stress could trigger inhibitory kappa B kinase, after which activated nuclear factor-kappa B (NF-κB) is dissociated [26]. Dissociated NF-κB induces the overexpression of TNF-α by binding to the promoter site of TNF-α. On the other hand, TNF-α could promote the production of inflammatory factor IL-6, which is a pro-hypertrophic factor that mediates the activation of the JAK2/STAT3 signaling pathway mediated by gp130 [30]. In this study, the expression of pro-inflammatory cytokines, TNF-α and IL-6 were significantly inhibited by LBPE (*p* < 0.01), indicating the anti-inflammatory effect of LBPE in hypertrophic cardiomyocytes. Furthermore, it could also be concluded that the inhibition of the JAK2/STAT3 signaling pathway could be mediated by IL-6.

It has already been confirmed that the stimulation of ISO can induce oxidative stress in cardiomyocytes, which leads to the production of reactive oxygen species (ROS), an oxygen free radical that can induce oxidative stress and attack cells [10]. ROS also activates various molecular pathways to injure myocardial cells indirectly [31]. In a recent study, cardiotrophin-1 inhibited the production of ROS in mouse cardiomyocytes, which can eliminate the activation of the JAK/STAT signaling pathway [32]. Additionally, the degree of oxidative stress injury of cells can be evaluated by the SOD activity as well the GSH and MDA content [4]. After ISO treatment, the content of MDA increased and the levels of SOD and GSH decreased, which means the antioxidant capacity was reduced. Since these indicators are regulated by LBPE, this suggests that LBPE might protect from CH by inhibiting oxidative stress.

Apoptosis is proposed as a potential target for treating CH and preventing HF [33]. Treatment with ISO caused excessive apoptosis of H9c2 cells induced by CH [34]. Constitutive STAT3 impacted mitochondrial respiration, which can release pro-apoptotic factors into the cytoplasm, causing apoptosis [27]. Currently, a study found that inhibition of JAK2/STAT3 signaling pathway could effectively prevent apoptosis and restrain CH [13]. Similar results were obtained in this study. We found that the expression of pro-apoptosis related factors decreased significantly while the anti-apoptosis related factor increased significantly after treatment with LBPE.

## 5. Conclusions

The present study demonstrates that LBPE can alleviate ISO-induced CH in H9c2 cardiomyocytes through inhibiting the JAK2/STAT3 signaling pathway mediated by IL-6. This research provides a basis for the development of lotus bee pollen in cardiovascular protection.

## Figures and Tables

**Table 1:** Primer sequences for quantitative real-time PCR.

Name	Accession No.	Forward Sequence (5’-3’)	Reverse Sequence (5’-3’)
*GAPDH*	NM_017008	AGCCCAGAACATCATCCCTG	ACGGATACATTGGGGGTAGG
*ANP*	NM_012612	GGGAAGTCAACCCGTCTCAG	CAATCCTACCCCCGAAGCAG
*BNP*	NM_031545	AGCTCTCAAAGGACCAAGGC	TCCGGTCTATCTTCTGCCCA
*β-MHC*	NM_017240	ATCAAGGGAAAGCAGGAAGC	CCTTGTCTACAGGTGCATCA
*Bax*	NM_017059	AGGATCGAGCAGAGAGGATG	AGCTCCATGTTGTTGTCCAGT
*Bcl-2*	NM_016993	GGGGCTACGAGTGGGATACT	GACGGTAGCGACGAGAGAAG
*Caspase-3*	NM_012922	CGGACCTGTGGACCTGAAAA	TAACCGGGTGCGGTAGAGTA
*Caspase-8*	NM_022277	CATCCTGACTGGCGTGAACT	TGGCATCTGCTTTCCCATGT
*Caspase-9*	NM_031632	GAGGATATTCAGCGGGCAGG	GCAGGAGATGAAGCGAGGAA
*IL-6*	NM_012589	TTCCAGCCAGTTGCCTTCTT	CTGGTCTGTTGTGGGTGGTA
*TNF-α*	NM_012675	TCGTAGCAAACCACCAAGCA	GGTGAGGAGCACATAGTCGG

**Table 2:** Effect of LBPE on SOD, GSH and MDA in H9c2 cells (n = 6).

Group	SOD (U∙mL^−1^)	GSH (µmol∙g prot^−1^)	MDA (nmol∙mg prot^−1^)
Negative control	25.6207 ± 2.9961 ^##^	40.1040 ± 5.4200 ^##^	0.9942 ± 0.1906 ^##^
ISO	15.8183 ± 1.1401	14.9001 ± 3.6260	1.6708 ± 0.0870
Positive	23.9008 ± 0.7505 ^##^	44.1822 ± 1.0518 ^##^	1.0640 ± 0.1397 ^##^
100 µg∙mL^−1^ LBPE	17.2687 ± 3.1454	17.4918 ± 6.4598	1.3534 ± 0.0919 ^#^
250 µg∙mL^−1^ LBPE	19.9866 ± 0.9251	27.1641 ± 11.6397	1.1742 ± 0.1502 ^##^
500 µg∙mL^−1^ LBPE	22.3155 ± 4.3936 ^##^	32.3704 ± 6.1971 ^#^	1.1179 ± 0.1311 ^##^

Note: compared with ISO group, # *p* < 0.05, ## *p* < 0.01.

## Data Availability

The data presented in this study are available on request from the corresponding author.

## Supplementary Materials

The following supporting information can be downloaded at: https://www.mdpi.com/article/10.3390/antiox12010088/s1, UPLC-QTOF MS analysis of LBPE; Table S1: Peak assignments for the analysis of LBPE.
