# Correction: Han et al. Lotus Bee Pollen Extract Inhibits Isoproterenol-Induced Hypertrophy via JAK2/STAT3 Signaling Pathway in Rat H9c2 Cells. *Antioxidants* 2023, *12*, 88

**DOI:** 10.3390/antiox14080934

**Published:** 2025-07-30

**Authors:** Shuo Han, Lifu Chen, Yi Zhang, Shihui Xie, Jiali Yang, Songkun Su, Hong Yao, Peiying Shi

**Affiliations:** 1 Department of Traditional Chinese Medicine Resource and Bee Products, College of Animal Sciences (College of Bee Science), Fujian Agriculture and Forestry University, Fuzhou 350002, China; 2 Department of Pharmaceutical Analysis, School of Pharmacy, Fujian Medical University, Fuzhou 350122, China; 3 State and Local Joint Engineering Laboratory of Natural Biotoxins, Fujian Agriculture and Forestry University, Fuzhou 350002, China

In the original publication [1], a mistake occurred in the 100 and 250 μg·mL^−1^ LBPE images in Figure 2A as published. The corrected Figure 2 appears below.

The authors state that the scientific conclusions are unaffected. This correction was approved by the Academic Editor. The original publication has also been updated.

## Figures and Tables

**Figure 2:**
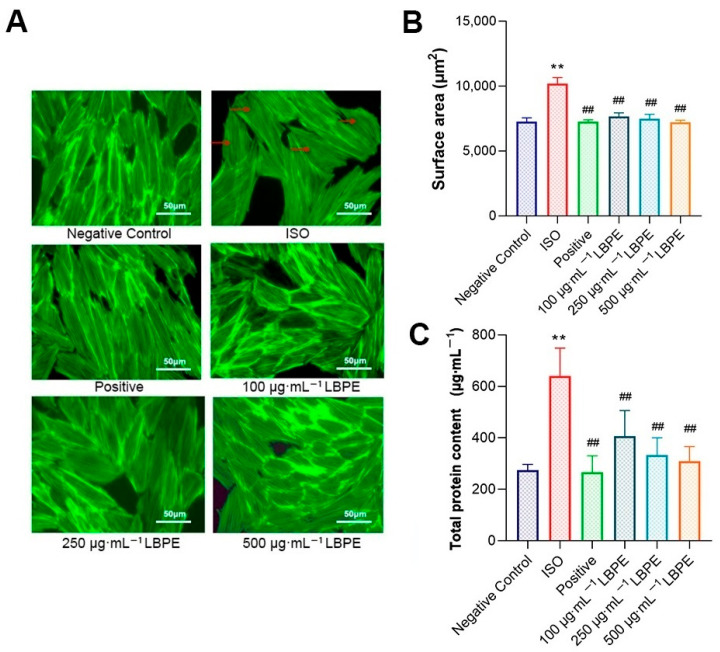
Effects of LBPE on the (**A**) morphology using phalloidin staining (×400), and the (**B**) surface area and (**C**) total protein content of H9c2 cardiomyocytes (*n* = 6). “→” indicates that the cells lose their spindle shape. ISO group vs. negative control group, ** *p* < 0.01. Positive and LBPE groups vs. ISO group, ## *p* < 0.01.
