# Supplementary material for: Lotus Bee Pollen Extract Inhibits Isoproterenol-Induced Hypertrophy via JAK2/STAT3 Signaling Pathway in Rat H9c2 Cells
Source: Antioxidants (Basel). 2022 Dec 30;12(1):88. doi: 10.3390/antiox12010088 (PMC9854735; doi:10.3390/antiox12010088)
Supplement: Supplementary file 1 [file antioxidants-12-00088-s001.zip › antioxidants-2071376-supplementary.pdf]

Supplementary Information

UPLC-QTOF MS analysis of LBPE

UPLC analysis was performed on an Agilent 1290 Infinity LC instrument (Agilent, Waldbronn, Germany) which consisted of a binary pump, a diode-array detector, an auto-sampler, and a column compartment. The samples were separated on a Zorbax SB-C_{18} column (3.5 μm, 100 mm × 2.1mm i.d.). The mobile phase was 0.1% formic acid aqueous solution (A) and methanol (B) after ultrasonic degassing, and the gradient elution conditions were as follows: 0-3 min: 10% (B); 3-10 min: 10%~65% (B); 10-10.5 min: 65~80% (B); 10.5~15 min: 80% (B). The UV detection wavelength was 254 nm. The column temperature and flow rate were respectively set at 20℃ and 0.2 mL/min, with a 1 μL volume of injection sample.

UPLC system connected with an Agilent 6530 QTOF mass spectrometer equipped with a dual ESI electrospray ion source. In the mode of (-) ESI, the capillary electrospray pressure was 3.5 kV; nebulizer pressure was 40 psig; drying gas (nitrogen) flow rate at 10.0 L/min; temperature set at 325 ℃ and OCT 1 RF Vpp at 750 V. The scanning range was *m/z* 100-1500.

Table S1 Peak assignments for the analysis of LBPE.

Peak No.	t_R (min)	Identification	(-) ESI-MS *m/z* Observed	Calculated (Δppm)	DBE	Formula
1	1.37	Gluconic acid	195.05066	195.05103 (-1.90)	1	$C_6H_{12}O_7$
2	2.23	Uridine[a]	243.06208	243.06226 (-0.74)	5	$C_9H_{12}N_2O$
3	2.78	Adenosine[a]	266.08942	266.08948 (-0.23)	7	$C_{10}H_{13}N_5$
4	3.48	Guanosine[a]	282.08431	282.08439 (-0.28)	7	$C_{10}H_{13}N_5$
5	11.72	Isorhamnetin 3-xylosyl-(1→2)-galactopyranoside	609.14676	609.14611 (1.07)	13	$C_{27}H_{30}O_{16}$

Note：[a] means compared with standard compounds.
